# Accessing the influence of ^99m^Tc-Sesta-MIBI-positive thyroid nodules on preoperative localisation studies in patients with primary hyperparathyroidism

**DOI:** 10.1007/s00423-022-02442-7

**Published:** 2022-01-21

**Authors:** Lindsay Hargitai, Maria Schefner, Tatjana Traub-Weidinger, Alexander Haug, Melisa Arikan, Christian Scheuba, Philipp Riss

**Affiliations:** 1 Endocrine Surgery, Division of Visceral Surgery, Department of General Surgery, Medical University of Vienna, Waehringer Guertel 18-20, Vienna, Austria; 2 Division of Nuclear Medicine, Department of Biomedical Imaging and Image-Guided Therapy, Medical University of Vienna, Waehringer Guertel 18-20, Vienna, Austria

**Keywords:** MIBI, Primary hyperparathyroidism, Parathyroidectomy, Thyroid nodule, Surgery

## Abstract

**Purpose:**

Curative treatment for primary hyperparathyroidism (PHPT) is parathyroidectomy (PTX) with removal of the hyperfunctioning gland(s). In an endemic goitre region, 35–78% of PHPT patients show concomitant thyroid disease. This study aimed to evaluate if ^99m^Tc-sestamibi (MIBI)-positive thyroid nodules decrease sensitivity in regard to localising the hyperfunctioning parathyroid gland(s) in PHPT patients.

**Methods:**

Within 5 years, 497 consecutive patients with biochemically proven PHPT were included in this study. The data was analysed retrospectively.

**Results:**

In total, 198 patients underwent PTX with thyroid surgery and 299 patients underwent sole PTX. Sensitivity of MIBI scan for PTX with and without thyroid surgery was 72.1% and 73.6%, respectively. A statistically significant difference in sensitivity of ultrasound for PTX with and without thyroid surgery (57.0% and 70.9%, respectively) was observed (*p* = 0.029). Thyroid nodule histology did not have a significant effect on the MIBI scan. Unilateral neck exploration (UNE) was performed in 110 patients and bilateral neck exploration (BNE) in 177 patients. The probability of surgical conversion from UNE to BNE due to incorrect localisation was 1.733 times higher in patients with thyroid nodules.

**Conclusions:**

Concomitant benign thyroid nodules did not influence MIBI sensitivity. No correlation between thyroid carcinoma and MIBI uptake was determined. However, MIBI detection of thyroid malignancy is important in patients initially being considered for minimal invasive parathyroidectomy. Sensitivity and positive predictive value of ultrasound were significantly lower in patients with thyroid nodules. The probability of conversion from UNE to BNE due to incorrect localisation was 1.733 times higher in patients with thyroid nodules.

## Introduction

Primary hyperparathyroidism (PHPT) is the third most common endocrine disease. Sporadic forms of PHPT, the most common forms of PHPT, are most frequently seen in women older than 50 years [1]. The only curative option is parathyroidectomy (PTX) [1]. In the literature, 15–84% of PHPT patients in endemic goitre regions have additional thyroid disease [2–6]. Interestingly, in 2–24% of patients with PHPT, thyroid carcinoma is diagnosed [7].

The most commonly employed preoperative localisation studies include neck ultrasound (US) and ^99m^Tc-sestamibi scintigraphy (MIBI). The overall accuracy of US is 88%, with a range between 76 and 87% and positive predictive value of 93–97% [8]. Preoperative US also plays an important role in the diagnosis of concurrent thyroid nodules that may necessitate biopsy before surgery or even simultaneous removal during the PTX. MIBI scintigraphy has a high accuracy of 97% with a high sensitivity of 90% in identifying and localising hyperfunctioning parathyroids [9]. The most common cause for false positive MIBI scans are solid thyroid nodules [10]. A delayed washout of the tracer is observed in thyroid carcinoma, reactive lymph nodes, lymph node metastasis, PTH-producing paragangliomas and enlarged submandibular saliva glands [11, 12]. False negative results are most commonly seen in multi-glandular disease or hyperplasia, as well as small adenomas [10, 12]. MIBI scintigraphy can be used in evaluating thyroid nodules in order to assess the risk of malignancy [13]. When used as a complementary diagnostic workup tool to fine needle biopsy or technetium-99 m thyroid scan in scintigraphically cold, suspicious thyroid nodules, MIBI can achieve a high specificity ranging from 78 to 100% and sensitivity of 55–91% [14, 15].

Several factors can decrease the sensitivity of MIBI in PHPT patients with concomitant thyroid nodules. Firstly, MIBI accumulation in thyroid nodules can lead to false positive results [4, 16]. Secondly, subtraction scintigraphy results that are not reliable can occur when thyroid uptake is reduced or suppressed, resulting in an increase in the number of negative localisation studies [2, 17, 18]. Some studies have investigated the impact of concomitant benign thyroid nodules on MIBI preoperative localisation of parathyroid adenomas; however, the results differ significantly. While some studies have determined the accuracy of MIBI in localising hyperfunctioning parathyroids to be substantially decreased [19, 20], others have shown no difference [16, 21]. In addition, all of these studies were conducted on small patient populations. This study aimed to investigate the impact of benign and malignant MIBI-positive thyroid nodules on sensitivity in terms of preoperative localisation of hyperfunctioning parathyroids in a large population of PHPT patients. Furthermore, the influence of malignant thyroid nodules on MIBI uptake was evaluated in terms of risk assessment.

## Materials and methods

### Patients

All patients with the biochemical diagnosis of PHPT who underwent surgical parathyroidectomy with/without thyroid surgery between 2009 and 2015 were included in this analysis.

Inclusion criteria were sporadic PHPT with available data regarding clinical symptomatology, preoperative US and MIBI scan, method of PTX, method of thyroid surgery, final histology and follow-up data.

### Preoperative localisation studies

All patients preoperatively underwent a standard double-phase MIBI scintigraphy with SPECT and high-resolution US with colour Doppler of the cervical region to localise one or more enlarged (presumed hyperfunctioning) parathyroid gland(s). US was also used to evaluate the morphology of the thyroid gland, in accordance to previously published standard protocols [22, 23]. All MIBI and US images and reports were retrospectively reviewed.

### Terminology according to Vienna Criteria [23]

True positive (TP): Removal of parathyroid gland with adequate decline of PTH and postoperative normocalcemia = correct prediction of complete resection.

False positive (FP): Removal of parathyroid gland with adequate decline of PTH and persistent hypercalcemia = incorrect prediction of complete resection.

True negative (TN): Removal of parathyroid gland with inadequate decline of PTH, thus requiring additional gland resection or operative failure and persistent hypercalcemia = correct prediction of incomplete excision.

False negative (FN): Removal of parathyroid gland with inadequate decline of PTH but postoperative normocalcemia = incorrect prediction of incomplete excision.

### Surgical procedure

The results of preoperative localisation determined surgical strategy, in accordance with the ESES consensus statement [22, 24]. Minimal invasive parathyroidectomy (MIP) (skin incision = 20–25 mm) was performed on the side of the predicted enlarged/hyperfunctioning single gland disease by MIBI, irrespective of US.

If the enlarged (hyperfunctioning) gland could not be localised at the presumed location or if intraoperative parathyroid hormone monitoring (IOPTH) was inadequate, the exploration was extended to a unilateral/bilateral exploration (UNE/BNE). In case of negative MIBI, surgery was carried out on the side of positive US. Patients with negative MIBI *and* US, and/or contralateral and/or bilateral thyroid pathology requiring surgery were selected for initial BNE. Criteria for concurrent thyroid surgery were suspicious unilateral or bilateral multinodular goitre or solitary nodules ≥ 5 mm that demonstrate US characteristics typical for malignancy, such as hypoechogenicity, microcalcifications, taller than wide shape or an ill-defined contour. In patients with additional thyroid surgery, a hemithyroidectomy or total thyroidectomy was performed after removing the enlarged parathyroid glands and after interpretation of IOPTH.

### Statistical analysis

Categorical variables are reported as absolute numbers and percentages, while continuous variables are given as mean with standard deviation. Sensitivity and positive predictive value (PPV) were calculated using 2 × 2 tables. Descriptive statistics analysed patient characteristics. Chi-quadrate tests and Fischer’s exact test were used to determine statistical significance. Analysis of data was performed using SPSS version 23 for Windows (Chicago, IL, USA).

## Results

### Patient characteristics and symptoms

Four hundred ninety-seven patients (female: *n* = 341 (69.8%); male: *n* = 156 (31.2%)) were included in this analysis. The average age was 60.2 (± 13.1) years.

In total, 153/497 (30.8%) patients were asymptomatic. Conversely, 228/497 (45.9%) patients showed symptoms of arterial hypertension, hypercalcemia, osteopenia, bone pain and depression. Typical symptoms, such as bone manifestations and renal manifestations, were observed in 69.2% and 20.3%, respectively. Depression was seen in 45.9% of patients, 0.6% presented with hypercalcaemic crisis and 0.2% had a brown tumour.

### Preoperative parathyroid US

In total, 318/497 (64.0%) patients showed ≥ 1 thyroid nodule in the preoperative US. Overall, 198/318 (62.3%) of these patients had concomitant thyroid surgery: 103/198 (52.0%) thyroidectomies and 95/198 (48.0%) hemi-thyroidectomies.

True positive US results were seen in 132/299 (44.1%) patients and 65/198 (32.8%) patients that underwent sole PTX and PTX with concomitant thyroid surgery, respectively. False positive results were observed in 113/299 (37.8%) and 84/198 (42.4%) patients that underwent sole PTX and PTX plus thyroid surgery, respectively. False negative results were seen in 54/299 (18.1%) patients with sole PTX and 49/198 (24.7%) patients with PTX and thyroid surgery. Statistical analysis showed a significant difference in the ability of preoperative US to more often correctly identify parathyroid adenomas in patients without thyroid nodules (*p* = 0.029; see Table 1).

**Table 1:** Preoperative ultrasound imaging. Categorical variables are reported as absolute numbers and percentages as well as 95% confidence intervals

	Sole PTX	PTX + thyroid surgery	Total	*p* value
TP	132 (44.1%)	65 (32.8%)	197 (39.6%)	0.029
FP	113 (37.8%)	84 (42.4%)	197 (39.6%)	-
FN	54 (18.1%)	49 (24.7%)	103 (20.7%)	-
Sensitivity	70.9% (95% CI: 63.88–77.38%)	57.0% (95% CI: 47.41–66.25%)	-	-
PPV	53.9% (95% CI: 51.55–56.19%)	43.6% (95% CI: 39.71–47.62%)	-	-

*PTX*, parathyroidectomy; *TP*, true positive; *FP*, false positive; *FN*, false negative; *PPV*, positive predictive value

In patients with concomitant thyroid nodules, US result was true positive in 20/65 (30.8%) patients with thyroid carcinoma and in 45/65 (69.2%) patients with benign thyroid nodules. In terms of false positive results, 25/84 (29.8%) patients had thyroid carcinoma and 59/84 (70.2%) patients had benign nodules. Finally, 12/49 (24.5%) patients had thyroid carcinoma, but false negative US results, and 37/49 (75.7%) patients had false negative US result, but benign thyroid nodule in the final histology. This difference was not statistically significant (*p* = 0.739).

### Preoperative parathyroid imaging with MIBI scan

In patients who underwent sole PTX, 170/299 (56.9%) patients had true positive MIBI results whereas 101/198 (51.0%) patients with concomitant thyroid surgery had true positive results. False positive results were seen in 68/299 (22.7%) and 58/198 (29.3%) patients that underwent sole PTX and PTX plus thyroid surgery, respectively. False negative results were observed in 61/299 (20.4%) patients with sole PTX and 39/198 (19.7%) patients with PTX and thyroid surgery. There was no significant statistical difference in the MIBI localisation of parathyroid adenoma in patients with concomitant thyroid nodules (*p* = 0.248; see Table 2).

**Table 2:** Preoperative MIBI imaging. Categorical variables are reported as absolute numbers and percentages as well as 95% confidence intervals

	Sole PTX	PTX + thyroid surgery	Total
TP	170 (56.9%)	101 (51.0%)	271 (54.5%)
FP	68 (22.7%)	58 (29.3%)	126 (25.3%)
FN	61 (20.4%)	39 (19.7%)	100 (20.1%)
Sensitivity	73.6% (95% CI: 67.41–79.16%)	72.1% (95% CI: 63.94–79.38%)	-
PPV	71.4% (95%CI: 69.72–73.08%)	63.5% (95%CI: 60.98–65.99%)	-

*PTX*, parathyroidectomy; *TP*, true positive; *FP*, false positive; *FN*, false negative; *PPV*, positive predictive value

Furthermore, in patients with concomitant thyroid nodules, MIBI result was true positive in 30/101 (29.7%) patients with thyroid carcinoma (27 patients had papillary thyroid microcarcinoma ranging from 1 to 10 mm) and in 71/101 (70.3%) patients with benign thyroid nodules. In terms of false positive results, 13/58 (22.4%) patients had thyroid carcinoma (eight patients had papillary microcarcinoma and one patient medullary microcarcinoma, all ranging between 0.3 and 10 mm) and 45/58 (77.6%) benign nodules. Lastly, 14/39 (35.9%) patients had thyroid carcinoma (11 patients had papillary microcarcinoma ranging from 0.4 to 2.5 mm), but false negative MIBI result, and 25/39 (64.1%) patients had false negative MIBI results, but benign thyroid nodule in the final histology. This difference was not statistically significant (*p* = 0.341; see Table 3).

**Table 3:** Preoperative MIBI imaging and thyroid carcinoma. Categorical variables are reported as absolute numbers and percentages as well as 95% confidence intervals

	Thyroid carcinoma	Benign nodules	Total
TP	30 (29.7%)	71 (70.3%)	101 (51.0%)
FP	12 (20.7%)	46 (79.3%)	58 (29.3%)
FN	14 (35.9%)	25 (64.1%)	39 (19.7%)
Sensitivity	68.18% (95% CI: 52.42–81.39%)	73.96% (95% CI: 64.00–82.38%)	-
PPV	69.77% (95%CI: 64.28–74.74%)	61.21% (95% CI: 58.17–64.16%)	-

*TP*, true positive; *FP*, false positive; *FN*, false negative; *PPV*, positive predictive value

### Surgical procedures

The most common surgical procedure conducted was MIP in 210/497 (42.3%) patients followed by BNE in 177/497 (35.6%) and UNE in 110/497 (22.1%) patients. Patients underwent concurrent thyroid surgery when suspicious unilateral or bilateral multinodular goitre(s) or solitary nodule(s) ≥ 5 mm demonstrated US characteristics typical for malignancy (described above in “Materials and methods”). This allowed for the exclusion and sufficient treatment of thyroid carcinoma. In total, 299/497 (60.2%) patients underwent sole PTX. The remaining 198/497 (39.8%) patients had PTX with concomitant thyroid surgery. Of these 198 patients, 95/198 (48.0%) underwent a hemithyroidectomy and 103/198 (52.0%) total thyroidectomy. In 37/198 (18.7%) patients, a conversion from UNE to BNE was required based on inadequate IOPTH decline and false preoperative localisation, whereas a conversion in patients with sole PTX was only observed in 35/299 patients (11.7%; see Table 4).

**Table 4:** Surgical procedures. Categorical variables are reported as absolute numbers and percentages

	Total	*p* value
Minimal invasive parathyroidectomy	210 (42.3%)	n.s
Unilateral neck exploration	110 (22.1%)	n.s
Conversion from unilateral neck exploration to bilateral neck exploration	37 (18.7%)	0.03
Bilateral neck exploration	177 (35.6%)	n.s
Sole parathyroidectomy	299 (60.2%)	n.s
Parathyroidectomy + thyroid surgery	198 (39.8%)	n.s

### Follow-up

#### Histology

The majority of patients, 495/497 (99.6%), were diagnosed with parathyroid adenoma and in 2/497 (0.4%) patients, parathyroid hyperplasia was identified. Thyroid carcinoma was identified in 56/497 (11.3%) patients: 46/56 (82.1%) had papillary thyroid microcarcinoma (TNM staging: pT1a: 40 patients; pT1am: 5 patients; pT1bm: 1 patient), 5/56 (8.9%) papillary thyroid carcinoma (TNM staging: pT1bm: 1 patient; pT1b: 1 patient; pT3: 1 patient; pT3b: two patients), 2/56 (3.6%) medullary thyroid microcarcinoma (TNM staging: pT1a in both patients), 1/56 (1.8%) medullary thyroid carcinoma (TNM staging: pT2a), 1/56 (1.8%) follicular thyroid carcinoma (TNM staging: pT2) and 1/56 (1.8%) insular thyroid carcinoma (TNM staging: pT2a).

## Discussion

### Patients and symptoms

The majority of patients (69.8%) suffering from PHPT had single adenomas or hyperplasia in the final histology, and are female and over the age of 60 years, similar to the literature [25, 26]. This study observed a rate of papillary thyroid (micro)carcinoma and PHPT of 10.3%, lying within the literature rates of 0.9–18.2% [27]. One must consider that the majority of the carcinomas in this study were papillary microcarcinomas, which has an incidence rate of up to 12% [28] and that Austria is an endemic goitre region.

In the literature, the rate of osteoporosis lies between 39 and 62.9% [29, 30]. The higher prevalence observed in this study could be influenced by bias as the diagnosis of osteoporosis may have led to the screening for PHPT. The frequency of depression observed in the study lies within the rate cited in the literature (30–62%). However, one must keep in mind that neuropsychiatric symptoms can be missed in the diagnosis of PHPT, especially in elderly patients [31, 32]. Kidney stones are typically seen in 10–20% of patients [33]. The slightly higher rate seen in this study might be related to the higher prevalence of osteopenia/osteoporosis, given that the calcium status of the bones influences the production of kidney stones [34].

### Preoperative imaging with ultrasound

According to the consensus statement of ESES, preoperative imaging should include MIBI scan as the initial test, but US by an experienced investigator can also be conducted [24]. In our institution, all patients undergo preoperative US and MIBI scan. In a study by Prager et al. [2], the combined sensitivity of US and MIBI scan was 98%.

In this study, 64.0% of patients had at least one thyroid nodule in preoperative US. While this rate is higher than in previous studies [35], in endemic goitre regions, the rate of PHPT with thyroid nodules ranges between 15 and 84% [2–6]. Therefore, preoperative US is so important in patients with PHPT [2, 3]. In this analysis, sensitivity and PPV of US lie within the literature range of 51–96% [1, 2]. A high rate of false negative patients contributed to the low sensitivity. Additionally, the low PPV of this analysis was due to the high rate of false positive results. While US is safe, less expensive, non-invasive and quick, its sensitivity can be decreased in cases of multiple parathyroid adenomas or parathyroid hyperplasia, as well as concomitant thyroid nodules [36, 37]. In addition, US is operator-dependent, and it can be difficult to discriminate between parathyroid adenoma, small thyroid nodules and lymph nodes [38].

In patients with concomitant thyroid nodules, the sensitivity and PPV of US were both very low with 57.0% and 43.6%, respectively, which has been observed in other studies [2, 39]. Statistical analysis showed a significant difference in preoperative US diagnostics between patients with and without thyroid nodules (*p* = 0.029). While this study showed no statistically significant difference between benign nodules and thyroid carcinoma in the preoperative US, sensitivity was decreased in patients with benign nodules versus those with thyroid carcinoma. Studies have investigated US and MIBI in PHPT patients with thyroid nodules. While Mazzeo et al. [40] showed no difference in the sensitivity between the two, several studies have demonstrated that the sensitivity of both US and MIBI was decreased in patients with concomitant thyroid nodules [11, 39], as was observed in this study.

### Preoperative imaging with MIBI

In MIBI imaging, thyroid nodules can result in a higher uptake without washout, leading to false positive and even false negative results [10]. The overall sensitivity of MIBI lies between 61 and 80% [41, 42]. The sensitivity in this analysis was 73.6%, which lies within the reported range. MIBI also had a high rate of false negative patients, which contributed to the relatively low sensitivity. PPV of MIBI was higher than with US. Size of the parathyroid adenoma and concomitant thyroid nodules are the most important factors influencing the PPV of MIBI [42]. Given that in an endemic goitre region more thyroid nodules are observed, this can explain the relatively low PPV in this analysis.

In patients with concomitant thyroid nodules, sensitivity and PPV were slightly lower than in patients without nodules. Statistical analysis showed no significant difference. A negative influence of thyroid lesions on MIBI sensitivity has also been observed in previous studies [20, 39]. Hyper-functional thyroid cells in benign lesions can cause false positive MIBI results [14]. The overall sensitivity and PPV of MIBI in benign thyroid nodules were 73.9% and 61.2%, respectively. In this study, 9.0% of patients had false positive MIBI results with benign thyroid nodules, 8.6% of patients nodular goitre in the final histology, 0.2% of patients Graves’ disease and 0.2% of patients thyroiditis. As with the study by Prager et al. [2], this study did not show a significant difference in the sensitivity and PPV of MIBI in patients with and without thyroid nodules.

### MIBI and thyroid carcinoma

Thyroid carcinoma is found in 1–36% of patients with PHPT [7, 14, 35]. In this study, 11.3% of patients had thyroid carcinoma in the final histology, lying within the reported literature range. Papillary microcarcinoma was the most common in 82.1% of patients, followed by papillary thyroid carcinoma in 8.9% of patients, 3.6% medullary microcarcinoma, 1.8% medullary thyroid carcinoma, 1.8% insular thyroid carcinoma and 1.8% follicular thyroid carcinoma.

The sensitivity of MIBI in patients with thyroid carcinoma observed in this study lies within the reported literature range of 55–91% [5, 14, 20, 35]. In this analysis, PPV was slightly higher than sensitivity. Both sensitivity and PPV did not show statistically significant differences between thyroid carcinoma and benign thyroid nodules. A meta-analysis by Treglia et al. [14] determined that MIBI is a sensitive diagnostic tool in the workup analysis of malignant thyroid nodules. However, it seems that MIBI uptake is not specific for thyroid carcinomas [14]. This analysis also found no correlation between histological type of thyroid carcinoma and MIBI uptake. Furthermore, there seems to be no correlation between MIBI uptake and thyroid nodule size [43]. In terms of false positive MIBI results, 22.4% of patients had thyroid carcinoma in the final histological report. Upon further retrospective analysis of the MIBI scans, five patients were MIBI positive, but the uptake was falsely identified as a parathyroid adenoma and not thyroid carcinoma. In an additional five patients, MIBI scan was positive for the same side as the thyroid carcinoma, but in a different location, and in two patients, thyroid carcinoma was on the opposite side of the positive MIBI scan. In one patient, multi-glandular disease could not be ruled out, but the location of the positive uptake correlated with the thyroid carcinoma. This study could also not determine a correlation between the histology of the thyroid carcinoma and its influence on MIBI uptake, similar to the meta-analysis by Treglia et al. [14]. In total, 15% of patients with microcarcinomas had false positive MIBI scans. However, in all of these patients, the correct side was diagnosed in the MIBI scan. Therefore, the presence of the small cancers did not interfere with the imaging to the same degree as the larger nodules (> 10 mm). Nevertheless, preoperative detection of occult thyroid malignancy in MIBI scan is important in patients initially only being considered for MIP.

There are several factors that can affect the sensitivity of an imaging technique, such as the level of expertise and variations in the patient population, but also if the institution is a low- or high-volume centre [44]. By using more than one imaging modality, a more accurate preoperative location can usually be diagnosed [42]. While the new techniques, such as PET/CT, are promising, it is associated with higher costs and is not available in low-volume centres. One must take into consideration that higher initial imaging costs may correlate with higher rates of intraoperative parathyroid localisation, thus subsequently lowering the overall costs of parathyroid treatment.

### Surgical procedures

The majority of operations conducted were MIP in 42.3% of patients, followed by BNE in 35.6% of patients and UNE in the remaining 22.1% of patients. Concomitant thyroid surgery was conducted in 39.8% of patients. Given that Austria is an endemic goitre region, this high rate in additional thyroid operations is not surprising. A survey conducted by the American Association of Endocrine Surgeons stated that 14–30% of parathyroid surgeons would plan for concomitant thyroid surgery, regardless of nodule size [45]. However, even in endemic goitre regions, the extent of resection should be individualized due to the higher morbidity associated with total thyroidectomy [46]. In the literature, overall morbidity and mortality rates of concomitant (hemi)-thyroidectomy are rather low [47]. Recurrent laryngeal nerve palsy occurs in 2.1–4.1% (permanent in 0.3–1.2%) [48–50] and hypoparathyroidism in 6.6–24% (permanent in 0–4.4%) [48, 51, 52]. On the other hand, a carcinoma rate of 6–39% has been reported in PHPT patients with concomitant thyroid nodules [4, 53, 54]. The main indication for concomitant thyroid surgery is exclusion and appropriate treatment of malignancy and national guidelines should be adhered to [55, 56]. While patients who receive concomitant thyroid operations have a higher risk for temporary recurrent laryngeal nerve palsy and hypoparathyroidism, the high rate of incidentally detected thyroid carcinoma in endemic goitre regions indicates thyroid surgery when thyroid nodules are detected in preoperative ultrasound [57].

The remaining 60.2% of patients underwent sole PTX. A conversion from UNE to BNE was required in 18.7% of patients when there were concomitant thyroid nodules, whereas a conversion in patients without additional thyroid surgery was only observed in 11.7%. This conversion rate proved to be statistically significant (*p* = 0.03). Furthermore, patients with false preoperative localisation and concomitant thyroid nodules had a 1.733 higher risk of intraoperative conversion from UNE to BNE.

## Conclusion

The sensitivity of MIBI scans was not influenced by concomitant thyroid nodules, regardless if they were benign or malignant. While no correlation between thyroid carcinoma and its influence on MIBI uptake was determined, preoperative MIBI detection of thyroid malignancy is important in patients initially being considered for MIP. Preoperative US demonstrated significantly lower sensitivity and PPV in patients with thyroid nodules than in those without. Surgical conversion rate from UNE to BNE due to incorrect localisation was 1.733 times higher in patients with thyroid nodules.

## Data Availability

All data was retrieved from patient chart review.
